# Supplementary material for: Spatial patterns of Hyalomma marginatum-borne pathogens in the Occitanie region (France), a focus on the intriguing dynamics of Rickettsia aeschlimannii
Source: Microbiol Spectr. 2024 Jul 16;12(8):e01256-24. doi: 10.1128/spectrum.01256-24 (PMC11302286; doi:10.1128/spectrum.01256-24)
Supplement: Supplemental material — Tables S1 to S3. [file spectrum.01256-24-s0001.pdf]

Table S1: Tick-borne pathogens primers and probes sequences used for detection confirmation by PCRs and qCPRs.

Pathogen	Targeted gene	PCR type		Amorce name	Sequence
Anaplasma / Ehrlichia	16S rRNA (1)	Nested	PCR1	EHR1	GAACGAACGCTGGCGGCAAGC
				EHR2	AGT AYC GRA CCA GAT AGC CGC
			PCR2	EHR3	TGCATAGGAATCTACCTAGTAG
				EHR2	AGT AYC GRA CCA GAT AGC CGC
	msp2 (2)	Conventionnal		903F	AGT TTG ACT GGA ACA CAC CTG ATC
				1024R	CTC GTA ACC AAT CTC AAG CTC AAC
Babesia / Theileria	18S rRNA (3)	Nested	PCR1	BTH 18S 1st F	GTGAAACTGCGAATGGCTCATTAC
				BTH 18S 1st R	AAGTGATAAGGTTCACAAAACTTCCC
			PCR2	BTH 18S 2nd F	GGCTCATTACAACAGTTATAGTTTATTTG
				BTH 18S 2nd R	CGGTCCGAATAATTCACCGGAT
	18S rRNA (4)	Conventionnal		BABGF2	GYY TTG TAA TTG GAA TGA TGG
				BABGR2	CCA AAG ACT TTG ATT TCT CTC
Rickettsia	Citrate synthase (5)	Conventionnal		Rsfg877	GGGGGCCTGCTCACGGCGG
				Rsfg1258	ATTGCAAAAAGTACAGTGAACA
	OmpB (6)	Nested	PCR1	Rc.rompB.4362p	GTCAGCGTTACTTCTTCGATGC
				Rc.rompB.4,836n	CCGTACTCCATCTTAGCATCAG
			PCR2	Rc.rompB.4,496p	CCAATGGCAGGACTTAGCTACT
				Rc.rompB.4,762n	AGGCTGGCTGATACACGGAGTAA

Table S2: Multivariate analyses of pathogen infection rates.

	Geographic cluster (n = 510)					Tick sex (n = 510)					Engorgement status (females only, n=233)						
	Hérault/Gard geographic cluster (n=187)		Aude/Pyrénées-Orientales cluster (n=323)		Significant difference	Male (n=277)		Female (n=233)		Significant difference	Unfed (n=110)		Semi-engorged (n = 49)		Fed (n=74)		Significant difference
Pathogen	Nb of positive samples	Infection rate (%) (± 95% CI)	Nb of positive samples	Infection rate (%) (± 95% CI)	Yes/No (χ^2; df; p-value)	Nb of positive samples	Infection rate (%) (± 95% CI)	Nb of positive samples	Infection rate (%) (± 95% CI)	Yes/No (χ^2; df; p-value)	Nb of positive samples	Infection rate (%) (± 95% CI)	Nb of positive samples	Infection rate (%) (± 95% CI)	Nb of positive samples	Infection rate (%) (± 95% CI)	Yes/No (χ^2; df; p-value
R. aeschlimannii	147	78.6 (72,7-84,5)	298	92.3 (89,3-95,2)	Yes (χ^2=4.1210; df=1; p-value=0.04235)	243	87.7 (83.8-91.6)	202	86,7 (82.3-91.1)	No (χ^2= 0.0025; df= 1; p-value= 0.95995)	98	89.1 (83.1-95)	42	85.7 (75.6-95.9)	62	83.8 (75.2-92.4)	No (χ^2= 1.1716; df= 2; p-value= 0.55666)
T. equi	15	8.0 (4.1-12.0)	32	9.9 (6.6-13.2)	No (χ^2= 0.3689; df= 1; p-value= 0.5436)	30	10.8 (7.1-14.5)	17	7.3 (3.9-10.7)	No (χ^2= 0.0216; df= 1; p-value= 0.8832)	2	1.8 (0-4.4)	4	8.2 (0.2-16.1)	11	14.9 (6.6-23.2)	Yes (χ^2= 8.8679; df= 2; p-value= 0.01187
Francisella-LE	177	94.7 (91.4-97.9)	316	97.8 (96.2-99.4)	No (χ^2= 2.7711; df= 1; p-value= 0.09598)	263	94.9 (92.4-97.5)	230	98.7 (97.3-100)	Yes (χ^2= 4.4239; df= 1; p-value= 0.03544)	110	100	47	95.9 (90.2-100)	73	98.6 (96.0-100)	No (χ^2= 0.0251; df= 2; p-value= 0.9875)
A. phagocytophilum	1	0.5 (0-1.6)	7	2.2 (0.6-3.8)	No (χ^2= 0.8844; df= 1; p-value = 0.347003)	2	0.7 (0-1.7)	6	2.6 (0.5-4.6)	No (χ^2= 0.5058; df= 2; p-value= 0.476966)	0	0	3	6.1 (0-13.1)	3	4.1 (0-8.7)	Not applicable
A. marginale	0	0	4	1.2 (0-2.5)	Not applicable	0	0	4	1.7 (0-3.4)	Not applicable	0	0	1	2.0 (0-6.1)	3	4.1 (0-8.7)	Not applicable

Counts, infection rates and statistical analyses for each variable and each pathogen are presented. For the analyse of the geographic cluster and tick sex, infection rates were calculated by taking into account the number of positive samples for each pathogen and each variable category and the total number of ticks (n=510). The engorgement status influence was analysed on females only (n=233). The number of ticks for each level of variables are indicated for information. « Not applicable » is indicated when no statistical analysis was conducted because of a too low positive sample number. Data were generated using the BioMark™ assay for *R. aeschlimannii*, *Francisella*-LE, *A. phagopcytophilum* and *A. marginale. T. equi* data were generated using the qPCR assay. df is the degree of freedom and X², the Chi-square value.

Table S3: Multivariate analyse of *R. aeschlimannii* and *T. equi* loads obtained by qPCR.

	Geographic cluster					Tick sex					Engorgement status						
	Hérault/Gard geographic cluster		Aude/Pyrénées-Orientales cluster		Significant difference	Male		Female		Significant difference	Unfed		Semi-engorged		Fed		Significant difference
Pathogen	Nb of positive samples	Mean load (genome. µL^{-1}) (± 95% CI)	Nb of positive samples	Mean load (genome.µL^{-1}) (± 95% CI)	Yes/No (χ²;df; p-value)	Nb of positive samples	Mean load (genome. µL^{-1}) (± 95% CI))	Nb of positive samples	Mean load (genome. µL^{-1}) (± 95% CI)	Yes/No (χ²;df; p-value)	Nb of positive samples	Mean load (genome.µL^{-1}) (± 95% CI)	Nb of positive samples	Mean load (genome. µL^{-1}) (± 95% CI)	Nb of positive samples	Mean load (genome. µL^{-1}) (± 95% CI)	Yes/No (χ²;df; p-value)
R. aeschlimannii (n=456)	153	2.0×10^5 (1.4×10^5 – 2.6×10^5)	303	7.6×10^5 ($6,5 \times 10^5$ - $8,6 \times 10^5$)	Yes (χ²= 6.5292; df= 1; p-value= 0.01061)	246	3.2×10^5 (2.7×10^5 – 3.8×10^5)	210	8.6×10^5 (7.1×10^5 – 1.0×10^6)	Yes (χ²= 15.6199; df= 1; p-value= 7.447×10^{-5}	101	7.5×10^5 (5.6×10^5 – 9.4×10^5)	45	6.9×10^5 (4.0×10^5 – 9.9×10^5	64	1.1×10^6 (8.4×10^5 – 1.4×10^6)	No (χ²= 0.0852; df= 2 ; p-value= 0.958298)
T. equi (n=47)	15	5.3 (2.9 – 7.7)	32	1.0×10^3 (0 – 3.1×10^3)	Yes (χ²= 5.4868; df= 1; p-value= 0.01916)	30	1.1×10^3 (0 – 3.3×10^3)	17	31.3 (0.2– 62.4)	No (χ²= 1.0765; df= 1; p-value= 0.29948)	2	4.3 (0 – 34.9)	4	10.2 (0 – 32.3)	11	43.9 (0 – 92.8)	No (χ²= 1.2562; df= 2 ; p-value= 0.5336)

Counts, mean loads and statistical analyses for each variable and each pathogen are presented. The mean loads were calculated by taking into account only positive samples for *R. aeschlimannii* (n=456) and *T. equi* (n=47). The engorgement status influence on the pathogens load was analysed on positive females for *R. aeschlimannii* (n=210) and *T. equi* (n=17). The df is the degree of freedom and X², the Chi-square value.

Supplementary references

1. Anderson BE, Greene CE, Jones DC, Dawson JE. Ehrlichia ewingii sp. nov., the etiologic agent of canine granulocytic ehrlichiosis. Int J Syst Bacteriol. avr 1992;42(2):299-302.

2. Drazenovich N, Foley J, Brown RN. Use of real-time quantitative PCR targeting the msp2 protein gene to identify cryptic Anaplasma phagocytophilum infections in wildlife and domestic animals. Vector Borne Zoonotic Dis Larchmt N. 2006;6(1):83-90.

3. Masatani T, Hayashi K, Andoh M, Tateno M, Endo Y, Asada M, et al. Detection and molecular characterization of Babesia, Theileria, and Hepatozoon species in hard ticks collected from Kagoshima, the southern region in Japan. Ticks Tick-Borne Dis. juin 2017;8(4):581-7.

4. Bonnet S, Jouglin M, Malandrin L, Becker C, Agoulon A, L'hostis M, et al. Transstadial and transovarial persistence of Babesia divergens DNA in Ixodes ricinus ticks fed on infected blood in a new skin-feeding technique. Parasitology. févr 2007;134(Pt 2):197-207.

5. Regnery RL, Spruill CL, Plikaytis BD. Genotypic identification of rickettsiae and estimation of intraspecies sequence divergence for portions of two rickettsial genes. J Bacteriol. mars 1991;173(5):1576-89.

6. Choi YJ, Jang WJ, Kim JH, Ryu JS, Lee SH, Park KH, et al. Spotted fever group and typhus group rickettsioses in humans, South Korea. Emerg Infect Dis. févr 2005;11(2):237-44.
